# QRS Detection Based on Improved Adaptive Threshold

**DOI:** 10.1155/2018/5694595

**Published:** 2018-03-15

**Authors:** Xuanyu Lu, Maolin Pan, Yang Yu

**Affiliations:** School of Data and Computer Science, University of Sun Yat-sen, Guangzhou Higher Education Mega Center, No. 132 Waihuan East Road, Guangzhou 510006, China

## Abstract

Cardiovascular disease is the first cause of death around the world. In accomplishing quick and accurate diagnosis, automatic electrocardiogram (ECG) analysis algorithm plays an important role, whose first step is QRS detection. The threshold algorithm of QRS complex detection is known for its high-speed computation and minimized memory storage. In this mobile era, threshold algorithm can be easily transported into portable, wearable, and wireless ECG systems. However, the detection rate of the threshold algorithm still calls for improvement. An improved adaptive threshold algorithm for QRS detection is reported in this paper. The main steps of this algorithm are preprocessing, peak finding, and adaptive threshold QRS detecting. The detection rate is 99.41%, the sensitivity (Se) is 99.72%, and the specificity (Sp) is 99.69% on the MIT-BIH Arrhythmia database. A comparison is also made with two other algorithms, to prove our superiority. The suspicious abnormal area is shown at the end of the algorithm and RR-Lorenz plot drawn for doctors and cardiologists to use as aid for diagnosis.

## 1. Introduction

Cardiovascular disease has been the largest threat to human life for decades, and millions of people died due to delayed treatment. Today, doctors and cardiologists diagnose cardiovascular disease mainly through electrocardiogram (ECG), precise ones of which are available. Nevertheless, with the rapid increase of data, how to achieve better automatic ECG analysis has become the key point. As the most significant information of ECG, QRS complex needs to be identified more precisely for better automatic ECG analysis.

In 1947, American physicist, Dr. Norman J. Holter, invented a continuous bioelectric recording technique. Since then, the dynamic ECG playback system (Ambulatory ECG) and Holter system have been created.

Holter system can record the long-term ECG information, and with the aid of computers, the automatic ECG analysis and diagnosis are made available.

QRS complex detection is the first and the most important step of ECG analysis. After the detection of QRS complex and the ECG analysis that follows, the cardiologists can diagnose cardiovascular disease.

QRS complex detection algorithm has been studied for decades, two major methods of study being hardware and software methods classified by the way of realization, of which hardware methods are, according to literature, far less flexible and convenient, so that software methods are under more discussion.

There have been various kinds of software methods, such as method based on mathematical model, method based on pattern recognition, method based on image recognition, method based on the wavelet transform, and method based on the neural network.

Engelse and Zeelenberg [1] suggested a filter algorithm based on filtration and amplifying of thresholds, which introduced the idea of noise detection, and Ligtenberg and Kunt [2] improved the denoising effect by using the FIR band-pass filter and low-pass differential phase to filter the signal. In 1985, Pan and Tompkins [3] suggested a simple but practical dual threshold detection algorithm and accomplished real-time detection, which made it the most classic paper.

After that, Trahanias et al. [4] suggested a method based on mathematical morphology whose application was limited because it required high pretreatment, followed by Ruha et al.'s [5] practical matching filter, which was a 15–40 Hz band-pass filter, to detect QRS wave for better signal-noise ratio. However, the method of pattern recognition is rarely used in practical applications due to noise sensitiveness. In recent years, the method based on wavelet transform raised public interest for its good time-frequency localization. And it performs well in detection. Li and Zheng [6] applied the wavelet transform to the detection of QRS complex, and Martínez et al. [7] also used the wavelet transform method for the R wave positioning and QRS region detecting. They both performed well in denoising and reached a high detection rate. Nevertheless, it was not suitable when it comes to portable ECG system because of its huge computational complexity. After that, with the popularization of machine learning and large data, the neural network algorithm once again comes into the public view. Xue et al. [8] suggested the neural network-based adaptive matching filter method, and Saini et al. [9] used KNN algorithm for QRS detection and achieved a high accuracy in 2013. But due to the large amount of computation and large occupation of space, it was not widely accepted.

The method of threshold detection has been widely used in dynamic QRS detection because of its clear thoughts, high speed, and small occupation of RAM. Most of them can be realized in real-time detection, and for portable, wearable, and wireless ECG systems, it is a very suitable algorithm but the accuracy of detection still needs further improvement [10].

Therefore, an improved adaptive threshold algorithm is introduced in this paper for higher accuracy, which is the main point of our work. The detection rate of this algorithm is enhanced while its speed is still fast. The last but not the least, it can also show the suspicious abnormal area and draw the RR-Lorenz plot at the end of the algorithm, which can aid the doctors and cardiologists to diagnose.

## 2. Overview of Algorithm

**Figure 1:**
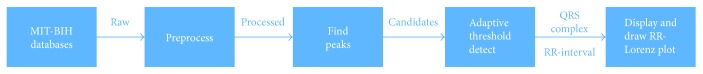
Schematic representation of intermediate steps for adaptive threshold algorithm implementation.

A schematic representation of intermediate steps for adaptive threshold algorithm implementation is drawn in Figure 1. In general, the overall detection process can be divided into four stages: (1) preprocessing, (2) peak finding, (3) adaptive threshold QRS detecting, and (4) displaying of abnormal area and drawing of RR-Lorenz plot. The tasks of QRS detection is performed and described as follows.

The principle of the threshold algorithm for QRS detection is that the QRS complex is the most characteristic band in ECG and has a high slope and apparent wave crest. Then, after concentrating this information, using a threshold to detect QRS complex becomes feasible. However, in the actual process, the first problem is that the ECG data collected from Holter device is noisy, which interferes with the detection a lot. There are three main types of noises: frequency interference, baseline drift, and EMG noise. Therefore, a preprocessing step for the data is necessary in order to remove the noise while focusing and amplifying the signals needed.

Preprocessing is basic and indispensable for this algorithm. Most noises mentioned above can be filtered out by using a band-pass filter because the main energy of QRS is in the range of 5–15 Hz. Then, the data is differentiated to figure out the highlight of the slope. However, the T wave also has a slope. To divide T and QRS waves, the feature is amplified by squaring and then this information is collected by window integration. After that, preprocessing step is completed.

The signals are integrated after the preprocessing step so that the detection is simplified, and here comes the crucial step: finding peaks. In this algorithm, peaks are regarded as the candidates of the QRS complex. When peaks are found, the nearby samples are checked to select the largest signal.

After finding all the candidates, the improved adaptive threshold algorithm is used to detect QRS complex. Firstly, an initial threshold is set, using the maximum signal of the first part of the sample. The threshold will adjust automatically according to the value of the candidate peaks in the process of detection, and there is a buffer to record the RR interval, which is used for the adaptive threshold. Because the heartbeat is continuous for a living person, when the algorithm does not find a QRS complex for a long time, it will adjust greatly.

When the detection is completed, it will display the suspicious risky arrhythmia location and draw the RR-Lorenz diagram to aid doctors and cardiologists to diagnose and give advice.

In this paper, we propose an improved adaptive threshold algorithm. In the algorithm, we put forward a peak-finding step for candidate selecting. Instead of using a barely local max as a candidate, a special method is applied. This step helps to find candidates of QRS waves more precisely and contributes a lot to QRS detection. And the threshold method is thus improved, which can adjust adaptively in the process of candidate selection. However, the threshold adjusts massively if it is thought to be in special conditions. The average of RR interval is also improved and is combined with time interval for condition judgment. Lastly, it will display the suspicious risky arrhythmia location and draw the RR-Lorenz diagram to aid doctors and cardiologists to diagnose and give advice.

## 3. Methodology

In this section, the proposed algorithm for the detection of QRS complex is described.

### 3.1. Preprocessing

Firstly, in order to attenuate noise, the signal passes through a digital band-pass filter composed of cascaded high-pass and low-pass filters. The desirable pass-band is in the range of 5–12 Hz. The following process is differentiation. The squaring process intensifies the slope of the frequency response curve of the derivative. The moving window integrator produces a signal that includes information of the slope and the width of the QRS complex [3].

**Figure 2:**
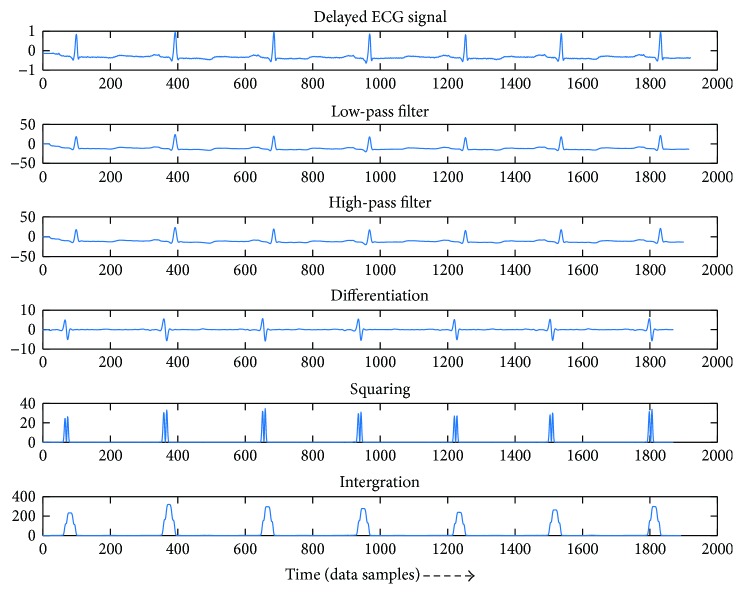
Preprocessing.

Figure 2 shows the process of preprocessing. After preprocessing, the desired energy is concentrated and amplified.

The following paragraphs describe the detail realizations and the principles of the steps of preprocessing.

#### 3.1.1. Low-Pass Filter

The difference equation of the filter is

(1) $$\begin{array}{c} y\left(nT\right)=2y\left(nT-T\right)-y\left(nT-2T\right)+x\left(nT\right)-2x\left(nT-6T\right)+x\left(nT-12T\right). \end{array}$$

Its delay is 6 sampling points.

#### 3.1.2. High-Pass Filter

The difference equation is

(2) $$\begin{array}{c} y\left(nT\right)=32x\left(nT-16T\right)-\left[y\left(nT-T\right)+x\left(nT\right)-x\left(nT-32T\right)\right]. \end{array}$$

The delay is 15 sampling points.

#### 3.1.3. Differentiation

The difference equation is

(3) $$\begin{array}{c} y\left(nT\right)=\left(\frac{1}{8}\right)\left[-x\left(nT-2T\right)-2x\left(nT-T\right)+2x\left(nT+T\right)+x\left(nT+2T\right)\right]. \end{array}$$

#### 3.1.4. Squaring

The squaring process amplifies the slope of the frequency response.

The difference equation is

(4) $$\begin{array}{c} y\left(nT\right)={\left[x\left(nT\right)\right]}^{2}. \end{array}$$

#### 3.1.5. Integration

The value is summed through a moving window with the width of 24 points (66.7 ms).

The difference equation is

(5) $$\begin{array}{c} y\left(nT\right)=\left(\frac{1}{N}\right)\left[x\left(nT-\left(N-1\right)T\right)+x\left(nT-\left(N-2\right)T\right)+\cdots +x\left(nT\right)\right]. \end{array}$$

### 3.2. Peak Detection

Here is a very critical step. When the preprocessing is completed, the following step is to determine the possible peaks as the candidates of the QRS complex. Read the input and record the most recent encountered maximum value. When the current value dropped to half of the last max value, it records the maximum value of a peak; if it does not find a peak in 300 record points, a local max value is selected. Finally, the peaks are checked and the largest value would be remained if the peak distance is less than 80 sampling points (222 ms).

**Figure 3:**
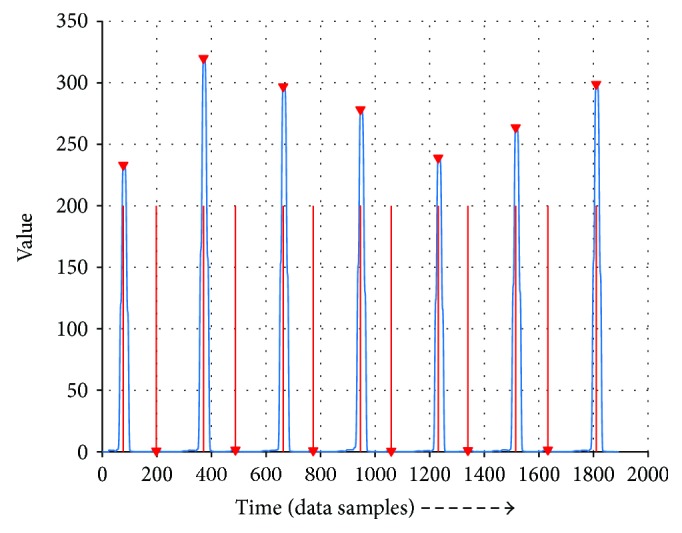
Finding peaks: the red lines show the positions, and the red marks show the integrated value of it.

Figure 3 shows the peaks that the algorithm detects, the red lines show their positions, and these peaks are the candidates of the QRS complex's position.

### 3.3. Threshold Detection

Firstly, initialize the threshold. The max of the first sampling point is to be found. It defines two kinds of peaks: signal peak and noise peak.

(6) $$\begin{array}{c} M\_VAL=\max\left(INPUT\left(1:300\right)\right),\\ SPK=0.13*M\_VAL,\\ NPK=0.1*SPK,\\ THRESHOLD=0.25*SPK+0.75*NPK, \end{array}$$

where INPUT is the signal after preprocessing, M_VAL is the max of the first 300 sampling point, SPK is the estimate of the signal peak, NPK is the noise peak, and THRESHOLD is the applied threshold.

Then the peaks, which are the candidates of QRS complex, are traversed. If a peak value is larger than the threshold, we mark it as a QRS complex, and then the SPK value is refreshed. Otherwise, we judge it as a noise peak and the NPK value is refreshed.

At the same time, the latest ten QRS complex is stored in a buffer and then the average RR interval of them is calculated. The RR interval is used to judge the state of the patient, because when the RR interval turns to be too large or too tight, it suggests that adjustment to the strategy is needed in time.

(7) $$\begin{array}{c} AVE\_RR=\frac{\left({RR}_{n-9}+{RR}_{n-8}+\cdots +{RR}_{n}\right)}{10}. \end{array}$$

If a QRS complex is not found in a long time, the threshold adjusts immediately because the heart rate is continuous. If it does not find a peak in 400 sampling points (1111 ms) or 1.5 times RR interval, it reduces the SPK value by half:

(8) $$\begin{array}{c} SPK=0.5*SPK. \end{array}$$

And then it refreshes the THRESHOLD and looks back from the last detected QRS complex. After traversing all the peaks, this step is finished.

**Figure 4:**
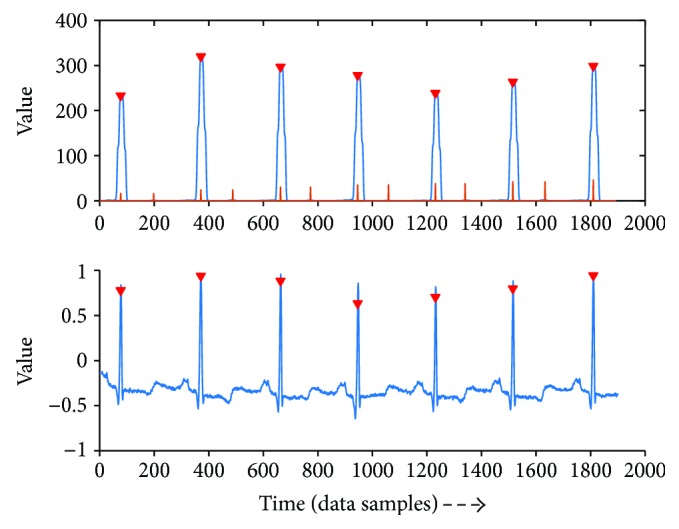
The detected QRS complex and the red line is the threshold value.

**Figure 5:**
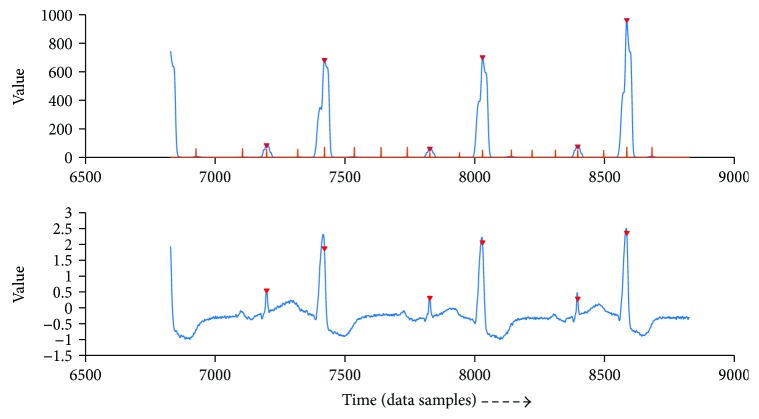
The threshold changes adaptively and finds the QRS complex.

Figure 4 shows the detected QRS complex and the variation of the threshold after each detected peak. If the peak value is larger than the threshold, it is marked as a QRS complex and otherwise as a noise peak. And the threshold is automatically adjusted.  Figure 5 shows that in the region of unstable heart rate, the threshold reduces sharply and finally finds the QRS complex.

### 3.4. Report Abnormal Region

When the algorithm does not find a QRS complex in 1000 ms, the RR interval is thought to be too large. It means the function of the heart may be suffering [11]. So, the region is displayed for the doctor or cardiologist to diagnose.

**Figure 6:**
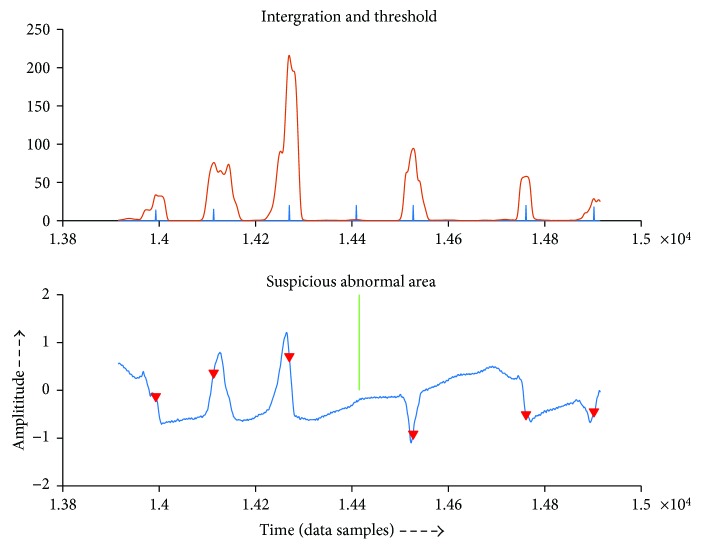
Example of reporting suspicious region.

**Figure 7:**
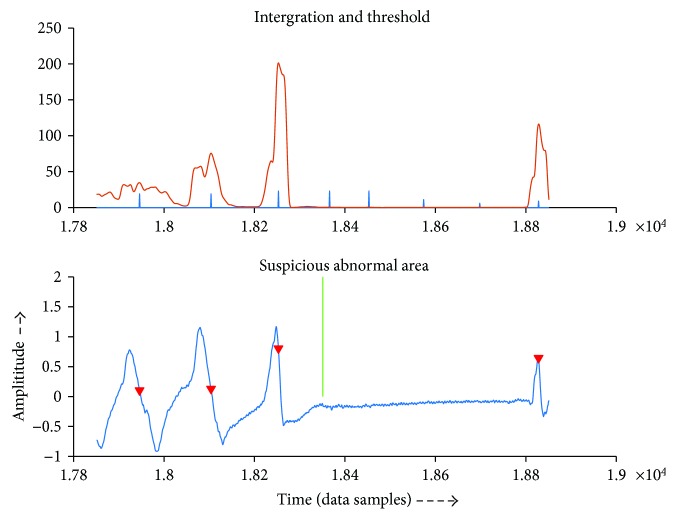
Example of reporting suspicious region.

Figures 6 and 7 show examples of displaying of suspicious abnormal region. The green line points to the region. The upper image shows the integration and thresh that algorithm detects to aid the doctor to analyze, and the lower one shows the original signal and the nearby QRS complex it detects.

### 3.5. RR-Lorenz Plot

After detection, the RR-Lorenz plot is drawn. It is the most used method in clinical practice. It reflects the variation of RR interval. The RR-Lorenz plot aids the doctor to diagnose [12].

(9) $$\begin{array}{c} X={RR}_{N},\\ Y={RR}_{\left(N+1\right)}. \end{array}$$

*X*-axle is the present RR interval; *Y*-axle is the next RR interval.

**Figure 8:**
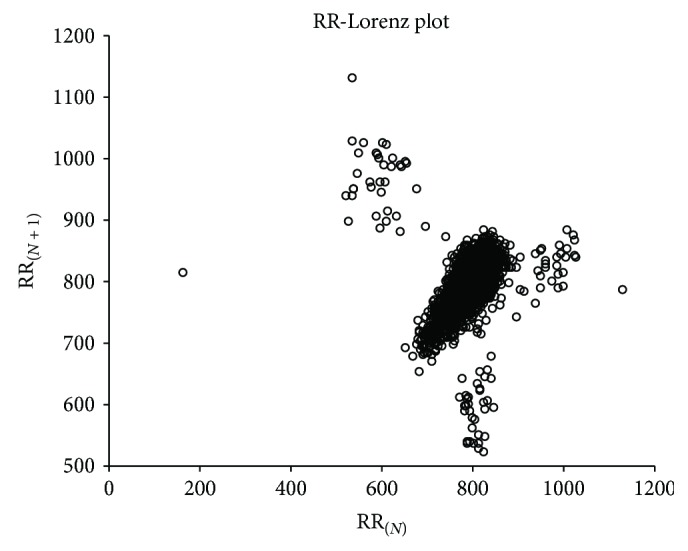
RR-Lorenz plot of record number 100 of MIT-BIH Arrhythmia database.

**Figure 9:**
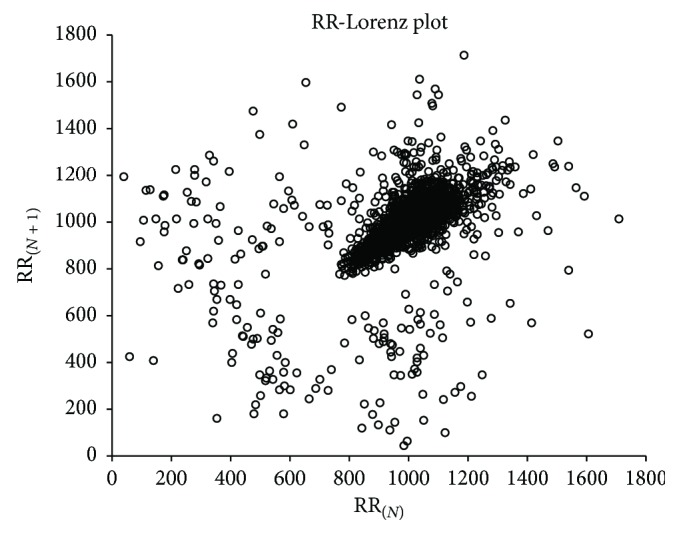
RR-Lorenz plot of record number 108 of MIT-BIH Arrhythmia database.

Figure 8 is record number 100 of the MIT-BIH, and Figure 9 is record number 108, in which we can find that the RR-Lorenz plot reflects the condition of the heart in a global view. The relatively normal record number 100 is much neater than record number 108.

## 4. Evaluation

The database used for algorithm detection is the classic MIT-BIH Arrhythmia database, which is the most commonly used QRS wave detection library. This library contains 48 half-hour records, a total of twenty-four hours of ECG data [13]. Each data is made up of two paths, the sampling frequency is 360 Hz, and two cardiologists have annotated all the beats, which contains more than 109,000 beats.

This algorithm used the MIT-BIH Arrhythmia database to evaluate this QRS detection algorithm. It uses MATLAB (R2015b) as the software in OS X EI Capitan of Macbook Pro. The CPU is 2.7 GHz Intel Core i5 with 8 G 1876 MHz DDR3 RAM.

Here, detection is said to be true positive (TP) and if the algorithm fails to identify QRS complex, it is called false negative (FN). If the algorithm detects non-QRS complex as QRS complex, it is thought to be false positive (FP).

(10) $$\begin{array}{c} Detection\;rate=\frac{\left(actual\;beats-failed\right)}{actual\;beats},\\ sensitivity\;\left(Se\right)=\frac{TP}{\left(TP+FN\right)},\\ specificity\;\left(Sp\right)=\frac{TP}{\left(TP+FP\right)}. \end{array}$$

The 24 h MIT-BIH database concludes more than 1–109000 beats; Table 1 shows the detection rate of the algorithm for MIT-BIH Arrhythmia database.

As Table 1 shows, for the actual 109966 beats, it successfully found 109653 beats. It has 337 FP and 313 FN. The detection rate is 99.41%, with Se = 99.72% and Sp = 99.69%.

Also, a comparison is made with two other real-time algorithms. And our algorithm performs well in detection rate, specificity, and sensitivity. The data is shown in Table 2.

In comparison, we compare our algorithm with two real-time algorithms. We first apply with the well-known Tompkins algorithm [3]. After preprocessing, it uses a dual-threshold method for QRS detection. But this algorithm does not perform well in record 108 with tall P waves and record 222 with baseline drift while our algorithm works better, especially in dealing with baseline drift. Chen et al. [14] used wavelet denoising for decision making. However, it failed to detect PVC's, for example, in record 208, but our algorithm detects most of them. Yet our algorithm does not perform very well with large number of supraventricular ectopic beats in record 232.

## 5. Conclusion

In this paper, QRS complex detection algorithm based on improved adaptive threshold method is proposed and validated with the MIT-BIH Arrhythmia database. Furthermore, the possibility of detecting QRS position of normal and abnormal QRS complex with influence of many artifacts is studied. In this work, we devoted to improved adaptive threshold algorithm. We also displayed the abnormal area and draw the RR-Lorenz plot for the doctor or the cardiologist to aid their diagnosis. The performance of the proposed algorithm gives the detection rate of 99.41%, sensitivity of 99.72%, and specificity of 99.69% on MIT-BIH Arrhythmia database. The algorithm is performing better in detecting beats with tall P waves and PVCs in comparison with traditional algorithm, which improved the specificity and the overall detection rate. With the advantages of threshold method's high-speed and small space occupation, this algorithm can be easily transplanted to portable, wearable, battery-operated, and wireless ECG systems.

## Figures and Tables

**Table 1:** Results of evaluating threshold algorithm using MIT-BIH Arrhythmia database.

Num	Actual	TP	FP	FN	Det_Rate
100	2273	2272	0	1	0.99956
101	1865	1865	3	0	0.99839
102	2187	2187	0	0	1.00000
103	2084	2082	1	2	0.99856
104	2229	2229	16	0	0.99282
105	2572	2572	44	0	0.98289
106	2027	2021	1	6	0.99655
107	2137	2132	1	5	0.99719
108	1763	1763	41	0	0.97674
109	2532	2527	1	5	0.99763
111	2124	2122	1	2	0.99859
112	2539	2538	1	1	0.99921
113	1795	1794	0	1	0.99944
114	1879	1879	6	0	0.99681
115	1953	1952	1	1	0.99898
116	2412	2401	3	11	0.99420
117	1535	1534	1	1	0.99870
118	2278	2277	1	1	0.99912
119	1987	1986	2	1	0.99849
121	1863	1861	1	2	0.99839
122	2476	2475	1	1	0.99919
123	1518	1514	1	4	0.99671
124	1619	1618	1	1	0.99876
200	2601	2600	2	1	0.99885
201	1963	1944	6	19	0.98726
202	2136	2128	3	8	0.99485
203	2980	2964	14	16	0.98993
205	2656	2651	1	5	0.99774
207	2332	2260	12	72	0.96398
208	2955	2945	3	10	0.99560
209	3005	3005	1	0	0.99967
210	2650	2601	3	49	0.98038
212	2748	2747	1	1	0.99927
213	3251	3243	0	8	0.99754
214	2262	2260	1	2	0.99867
215	3363	3359	1	4	0.99851
217	2208	2203	1	5	0.99728
219	2154	2152	2	2	0.99814
220	2048	2047	1	1	0.99902
221	2427	2404	1	23	0.99011
222	2483	2479	2	4	0.99758
223	2605	2583	1	22	0.99117
228	2053	2053	10	0	0.99513
230	2256	2255	1	1	0.99911
231	1571	1570	1	1	0.99873
232	1780	1780	139	0	0.92191
233	3079	3071	1	8	0.99708
234	2753	2748	1	5	0.99782
48	109966	109653	337	313	0.99409

**Table 2:** Comparison with other algorithm.

Method	Se (%)	P+ (%)	Er (%)
Our algorithm	99.72	99.69	0.59
Pan and Tompkins [3]	99.75	99.54	0.71
Chen et al. [14]	99.47	99.54	0.98

## References

[1] Engelse W. A. H., Zeelenberg C. (1979). A single scan algorithm for QRS-detection and feature extraction. *Computers in Cardiology*.

[2] Ligtenberg A., Kunt M. (1983). A robust-digital QRS-detection algorithm for arrhythmia monitoring. *Computers and Biomedical Research*.

[3] Pan J., Tompkins W. J. (1985). A real-time QRS detection algorithm. *IEEE Transactions on Biomedical Engineering*.

[4] Trahanias P., Skordalakis E., Papaconstantinou G. (1989). A syntactic method for the classification of the QRS patterns. *Pattern Recognition Letters*.

[5] Ruha A., Sallinen S., Nissilä S. (1997). A real-time microprocessor QRS detector system with a 1-ms timing accuracy for the measurement of ambulatory HRV. *IEEE Transactions on Biomedical Engineering*.

[6] Li C., Zheng C. QRS detection by wavelet transform.

[7] Martínez J. P., Almeida R., Olmos S., Rocha A. P., Laguna P. (2004). A wavelet-based ECG delineator: evaluation on standard databases. *IEEE Transactions on Biomedical Engineering*.

[8] Xue Q., Hu Y. H., Tompkins W. J. (1992). Neural-network-based adaptive matched filtering for QRS detection. *IEEE Transactions on Biomedical Engineering*.

[9] Saini I., Singh D., Khosla A. (2013). QRS detection using *K*-nearest neighbor algorithm (KNN) and evaluation on standard ECG databases. *Journal of Advanced Research*.

[10] Elgendi M., Eskofier B., Dokos S., Abbott D. (2014). Revisiting QRS detection methodologies for portable, wearable, battery-operated, and wireless ECG systems. *PLoS One*.

[11] Jin-Yi X., Yan-Yan Q., Qiong C., Qing-Yi W., Yao-Han W. (2014). Analysis of RR-Lorenz plot in patients of sinus rhythm with long RR interval. *Chinese Circulation Journal*.

[12] Yokota S., Yanagi H., Yuza N., Suzuki M., Nishida T. (1993). Evaluation of arrhythmias by Lorenz plot analysis on RR interval data from Holter ECG recordings. *The Japanese Journal of Medical Technology*.

[13] Massachusetts Institute of Technology MIT-BIH ECG database. http://www.physionet.org/cgi-bin/atm/ATM.

[14] Chen S. W., Chen H. C., Chan H. L. (2006). A real-time QRS detection method based on moving-averaging incorporating with wavelet denoising. *Computer Methods and Programs in Biomedicine*.

